# Resting heart rate is associated with colorectal advanced adenoma

**DOI:** 10.1371/journal.pone.0254505

**Published:** 2021-07-09

**Authors:** Jihye Park, Jong Soo Han, Hyun Jin Jo, Hyun Young Kim, Hyuk Yoon, Cheol Min Shin, Young Soo Park, Nayoung Kim, Dong Ho Lee

**Affiliations:** 1 Department of Internal Medicine, Institute of Gastroenterology, Yonsei University College of Medicine, Seoul, Korea; 2 Health Promotion Center, Seoul National University Bundang Hospital, Seoul National University College of Medicine, Seongnam, Korea; 3 Department of Internal Medicine, Seoul National University Bundang Hospital, Seongnam, Korea; Universidad Miguel Hernandez de Elche, SPAIN

## Abstract

**Background and aims:**

Resting heart rate is an independent predictor of colorectal cancer (CRC) development and CRC-related mortality. However, little is known about the relationship between resting heart rate and colorectal adenoma development. We aimed to investigate this association in a population who underwent screening colonoscopy.

**Methods:**

Among 39,021 patients who underwent both electrocardiogram and screening colonoscopy during routine health examinations at the Seoul National University Bundang Hospital, Health Promotion Center, Korea from January 2014 to July 2019, 1,344 patients had advanced adenoma. We performed 1:1 propensity score (PS) matching to establish a control group that mitigated the confounding effects of age and sex. We performed multivariate logistic regression analyses to identify the independent risk factors of advanced adenoma development.

**Results:**

Resting heart rate was significantly higher in the advanced adenoma group than in the control group. The prevalence of advanced polyp increased across the quartiles of resting heart rate. Patients with higher resting heart rates were more likely to be older, smokers, and have increased blood pressure and DM and less likely to engage in active exercises than those with lower resting heart rates. Patients with higher resting heart rates had higher serum glucose, triglyceride, hemoglobin A1C, and insulin levels and lower high-density lipoprotein cholesterol levels. Patients with resting heart rate in the highest quartile (≥71 bpm) still showed significantly increased odds ratio (OR) of advanced adenoma development (OR: 1.379, 95% confidence interval: 1.099–1.731, *p* = 0.006).

**Conclusions:**

High resting heart rate was a meaningful independent risk factor of advanced adenoma development.

## Introduction

Colorectal advanced adenoma is defined as a colorectal polyp with a diameter >1 cm, or presence of ≥3 adenomas per patient, and/or villous component, and/or severe dysplasia, predicting an increased likelihood of malignant transformation [1]. The prevalence of advanced adenoma ranged from 2.5% to 9.7% in the normal population [2]. Advanced adenoma has been regarded as a surrogate for colorectal cancer (CRC) in the adenoma-carcinoma pathway. Screening colonoscopy is recommenced for identifying and removing adenomas, particularly advanced adenomas. The development of CRC could be prevented by identifying the risk factors for advanced adenoma and actively performing screening colonoscopy for high-risk patients. Advanced adenoma development is strongly associated with old age, male sex, family history of CRC, cigarette smoking, obesity, and hyperglycemia in the previous studies [3–5].^.^

Resting heart rate has been suggested to be an important predictor of all-cause mortality, cardiovascular mortality, and cancer mortality [6–9]. Resting heart rate is an independent predictor of death in patients with CRC, pancreatic cancer, and non-small cell lung cancer [10]. Although the resting heart rate was not related to the overall cancer incidence in patients with vascular disease in a previous study, an increasing trend in resting heart rate was seen in patients with CRC development (hazard ratio [HR]: 1.19, 95% confidence interval [CI]: 1.00–1.42) [11]. Moreover, patients with CRC have a significantly higher resting heart rate than the cancer-free controls [12]. Moreover, several studies have demonstrated the protective effect of beta-blockers against cancer progression [13,14]. However, little is known about the relationship between resting heart rate and colorectal adenoma development.

A previous study reported a high resting heart rate associated with a significantly increased risk for advanced adenoma recurrence as detected by surveillance colonoscopy in CRC patients [15]. However, the study had a relatively small sample size and few cases of advanced adenoma recurrence (n = 27). Moreover, the resting heart rate was measured at the outpatient visit in a sitting position in the previous studies [12,15]. The present study aimed to assess the relationship between resting heart rate measured by an electrocardiogram (ECG) and advanced adenoma development in a population who underwent screening colonoscopy.

## Methods

### Patients and study design

We analyzed 39,021 patients who underwent both ECG and screening colonoscopy during routine health examinations at the Seoul National University Bundang Hospital, Health Promotion Center, Korea from January 2014 to July 2019. We excised all polyps detected during colonoscopy, and all specimens were sent to the pathology department for histological evaluation. Colorectal advanced adenoma was defined as a colorectal polyp with a diameter of >1 cm, or presence of ≥3 adenomas per patient, and/or villous component, and/or severe dysplasia based on the pathology specimens. A total of 1,344 patients had advanced adenoma in the present study. The study population was composed of 1,016 men (75.6%) and 328 women (24.4%). We performed 1:1 propensity score (PS) matching to establish control group without colon polyps that mitigated the confounding effects of age and sex. The exclusion criteria were as follows: patients with (i) incomplete medical records, (ii) a history of familial polyposis syndrome or Lynch syndrome, and (iii) known inflammatory bowel disease (IBD). This study was approved by the Institutional Review Board of Seoul National University Bundang Hospital, and the requirement for obtaining written informed consent from the patients was waived.

### Data collection

Resting heart rate was assessed by 12-lead ECG in a supine position after >10 min of sufficient rest in all patients at the time of screening colonoscopy. Blood pressure was measured in a sitting position using an upper arm cuff oscillometric blood pressure device. The patients were instructed to avoid eating, drinking alcohol, smoking, exercising, and bathing for 30 min before the measurement. Height and weight were measured by trained nurses. Body mass index (BMI) was calculated as weight in kilograms divided by the square of height in meters. The BMI was categorized following the World Health Organization (WHO) Asia-Pacific classification as normal (BMI <25.0 kg/m^2^) and obese (BMI ≥25 kg/m^2^) [16]. Data on physical activity, alcohol consumption, smoking status, and family history of CRC were obtained using self-reported questionnaires. Hypertension (HTN), diabetes mellitus (DM), dyslipidemia, coronary artery disease, peripheral artery disease, cerebrovascular disease, and arrhythmia were defined as the use of medications or “diagnosed by a physician”. Blood sampling was obtained the morning after an overnight fast. Plasma glucose, total cholesterol, triglyceride (TG), low-density lipoprotein (LDL) cholesterol, high-density lipoprotein (HDL) cholesterol, hemoglobin A1C (HbA1C), and insulin levels were obtained.

### Statistical analyses

The means and standard deviations or medians and ranges were calculated for all continuous variables, as appropriate. Categorical variables were expressed as proportions (%), and statistical analyses were performed to compare the variables between groups. Independent two-sample *t*-test or one-way analysis of variance (ANOVA) tests were used to compare continuous variables. Chi-square tests were used for categorical variables, as appropriate. We conducted an univariable logistic regression analysis for all variables. The variables which achieved a p-value < 0.05 after univariable logistic regression analysis were exported to the multivariable logistic regression analysis. Multivariate logistic regression analyses were carried out to identify the independent risk factors of advanced adenoma development, with adjustment for various confounders, including age, sex, alcohol history, smoking history, family history of CRC, exercise, BMI, HTN, DM, dyslipidemia, coronary artery disease, peripheral artery disease, cerebrovascular disease, arrhythmia, systolic blood pressure, and diastolic blood pressure. All statistical analyses were performed using the Statistical Package for Social Sciences (SPSS version 23.0; SPSS Inc., Armonk, NY, USA). The PS matching to establish control group without colon polyps that mitigated the confounding effects of age and were performed by R package (version 3.2.2). A *p-*value of <0.05 was considered statistically significant. All authors had access to the study data and reviewed and approved the final manuscript.

## Results

### Baseline characteristics

Screening colonoscopy was performed on 39,021 patients from January 2014 to July 2019 at the Seoul National University Bundang Hospital, Health Promotion Center, Korea. Of the t39,021 patients, 19,826 (50.8%) patients had colorectal polyps. Moreover, 1,344 (3.4%) patients had advanced adenomas.

The baseline characteristics of the advanced adenoma patients (n = 1344) and PS-matched controls (n = 1344) were investigated (**Table 1**). The advanced adenoma group had a higher proportion of patients who were smoking, engaging in less physical activity, with a higher BMI of >25 kg/m^2^, and with HTN, DM, peripheral artery disease, and cerebrovascular disease. Regarding the metabolic parameters, serum glucose, TG, hemoglobin A1C, and insulin levels were higher, and serum HDL cholesterol levels were lower in the advanced adenoma group than in the control group.

**Table 1:** Baseline characteristics of controls and advanced adenoma patients after 1:1 propensity score matching for age and gender.

Variables	Control group (n = 1344)	Advanced adenoma group (n = 1344)	*p value
Age (years)	56.8 ± 9.8	56.8 ± 9.8	0.970
Males	1017 (75.7%)	1016 (75.6%)	1.000
Smoking history	498 (37.1%)	584 (43.5%)	0.001
Alcohol history	600 (44.6%)	614 (45.7%)	0.614
Family history of colorectal cancer	78 (5.8%)	78 (5.8%)	1.000
Active exercise	443 (33.0%)	392 (29.2%)	0.037
BMI (≥ 25kg/m^2^)	421 (35.2%)	569 (44.6%)	< 0.001
Systolic blood pressure (mmHg)	119.7 ± 11.6	120.4 ± 12.5	0.190
Diastolic blood pressure (mmHg)	74.1 ± 9.1	74.3± 9.7	0.599
**Medical history**			
HTN	359 (26.7%)	412 (30.7%)	0.027
DM	135 (10.0%)	197 (14.7%)	< 0.001
Dyslipidemia	368 (27.4%)	405 (30.1%)	0.125
Coronary artery disease	56 (4.2%)	47 (3.5%)	0.422
Peripheral arterial disease	2 (0.1%)	12 (0.9%)	0.013
Cerebrovascular disease	13 (1.0%)	33 (2.5%)	0.004
Arrhythmia	9 (0.7%)	8 (0.6%)	1.000
**Metabolic parameters**			
Serum glucose (mg/dL)	95.1 ± 19.5	99.8 ± 29.2	< 0.001
Total cholesterol (mg/dL)	196.6 ± 39.1	199.1 ± 63.4	0.223
Serum triglyceride (mg/dL)	109.8 ± 71.3	125.3 ± 91.8	< 0.001
Serum high-density lipoprotein (mg/dL)	53.4± 12.1	51.9 ± 12.6	0.002
Serum low-density lipoprotein (mg/dL)	120.9 ± 31.7	121.1 ± 32.0	0.902
Hemoglobin A1C (%)	5.8 ± 1.7	5.9 ± 0.9	0.045
Serum insulin	7.4 ± 3.6	8.4 ± 4.4	< 0.001

Variables are expressed as mean ± SD or n (%).

*p value when comparing the lower resting heart rate group with the higher resting heart rate group.

b.p.m., beats per minute; BMI, body mass index; DM, diabetes mellitus; SD, standard deviation.

### Baseline characteristics according to the resting heart rates

The baseline characteristics of the study participants according to quartiles of the resting heart rate are shown in **Table 2**. Patients with higher resting heart rates were more likely to be younger (*p* trend = 0.020), to be smokers (*p* trend = 0.040), to engage in less active exercise (*p* trend = 0.001), to have higher systolic blood pressure (*p* trend = 0.027), to have higher diastolic blood pressure (*p* trend < 0.001), and to have DM (*p* trend < 0.001).

**Table 2:** Baseline characteristics of the study participants according to resting heart rates quartiles.

Advanced adenoma	Quartile 1 34–58 b.p.m (n = 642)	Quartile 2 59–64 b.p.m (n = 665)	Quartile 3 65–70 b.p.m (n = 654)	Quartile 4 71–138 b.p.m (n = 727)	*p value
Age (≥ 60 years)	259 (40.3%)	254 (26.0%)	200 (20.5%)	263 (26.9%)	0.020
Males	489 (76.2%)	490 (73.7%)	478 (73.1%)	576 (79.2%)	0.198
Smoking history	252 (39.3%)	256 (38.5%)	246 (37.8%)	3327 (45.0%)	0.040
Alcohol history	289 (45.0%)	301 (45.3%)	283 (43.3%)	341 (46.9%)	0.632
Family history of colorectal cancer	35 (5.5%)	37 (5.6%)	38 (5.8%)	46 (6.3%)	0.467
Active exercise	228 (35.5%)	217 (32.6%)	184 (28.1%)	206 (28.3%)	0.001
BMI (≥ 25kg/m^2^)	234 (40.0%)	236 (38.1%)	230 (38.4%)	290 (43.4%)	0.199
Systolic blood pressure (mmHg)	119.2 ± 11.8	119.4 ± 11.6	120.1 ± 12.4	120.2 ± 12.1	0.027
Diastolic blood pressure (mmHg)	72.7 ± 9.2	73.9 ± 9.0	74.5 ± 9.5	75.6 ± 9.8	< 0.001
**Medical history**					
Hypertension	184 (23.9%)	174 (22.6%)	182 (23.6%)	231 (30.0%)	0.134
DM	68 (10.6%)	72 (10.8%)	77 (11.8%)	115 (15.8%)	0.003
Dyslipidemia	178 (27.7%)	185 (27.8%)	178 (27.2%)	232 (31.9%)	0.108
Coronary artery disease	30 (4.7%)	26 (3.9%)	20 (3/1%)	27 (3.7%)	0.277
Peripheral arterial disease	0 (0.0%)	4 (0.6%)	2 (0.3%)	8 (1.1%)	0.014
Cerebrovascular disease	15 (2.3%)	6 (0.9%)	15 (2.3%)	10 (1.4%)	0.501
Arrhythmia	6 (0.9%)	3 (0.5%)	2 (0.3%)	6 (0.8%)	0.783
**Metabolic parameters**					
Serum glucose (mg/dL)	94.7 ± 20.8	95.5 ± 18.1	97.8 ± 29.1	101.2 ± 28.9	< 0.001
Total cholesterol (mg/dL)	198.2 ± 81.4	197.8 ± 38.2	197.7 ± 38.9	197.7 ± 41.7	0.998
Serum triglyceride (mg/dL)	106.6 ± 60.5	112.7 ± 67.1	123.6 ± 102.1	90.8 ± 3.4	< 0.001
Serum high-density lipoprotein (mg/dL)	53.8 ± 13.2	53.1 ± 12.0	52.6 ± 12.6	51.3 ± 11.7	0.002
Serum low-density lipoprotein (mg/dL)	119.7 ± 30.3	121.5 ± 30.4	121.9 ± 31.5	121.1 ± 34.7	0.654
Hemoglobin A1C (%)	5.8 ± 1.3	5.7 ± 0.6	5.7 ± 0.8	6.0 ± 2.2	< 0.001
Serum insulin	7.3 ± 3.7	7.8 ± 4.3	8.0 ± 4.0	8.4 ± 4.3	0.002

Variables are expressed as mean ± SD or n (%).

*p value when comparing quartile groups based on resting heart rates.

b.p.m., beats per minute; BMI, body mass index; DM, diabetes mellitus; SD, standard deviation.

When the metabolic parameters among the groups are compared according to the resting heart rate, significant differences in serum glucose (*p* trend< 0.001), TG (*p* trend < 0.001), HDL-C (*p* trend = 0.002), HbA1C (*p* trend < 0.001), and insulin levels (*p* trend = 0.002) were observed among the groups (**Table 2**). No significant difference was seen for total cholesterol and LDL-C levels.

### Advanced adenoma development rates according to the resting heart rates

Resting heart rate was significantly higher in the advanced adenoma group than in the control group (66.6 ± 10.4 vs. 65.0 ± 10.0 bpm, *p* < 0.001). The prevalence of advanced polyp showed an increasing tendency across the quartiles of resting heart rate (47.2% vs. 47.1% vs. 47.9% vs. 57.1%; *p* < 0.001; **Table 3**).

**Table 3:** Advanced adenoma development rates according to resting heart rates.

	Quartile 1 34–58 b.p.m (n = 642)	Quartile 2 59–64 b.p.m (n = 665)	Quartile 3 65–70 b.p.m (n = 654)	Quartile 4 71–138 b.p.m (n = 727)	*p* trend
Event	303 (47.2%)	313 (47.1%)	313 (47.9%)	415 (57.1%)	< 0.001
Univariate	1.000 (reference)	0.995 (0.801–1.236)	1.027 (0.826–1.277)	1.488 (1.202–1.842)*	
Age-adjusted	1.000 (reference)	0.995 (0.801–1.237)	1.028 (0.826–1.279)	1.489 (1.202–1.844)*	
Multivariable-adjusted^a^	1.000 (reference)	0.971 (0.773–1.221)	0.981 (0.779–1.236)	1.379 (1.099–1.731)*	

Variables are expressed as n (%).

b.p.m., beats per minute.

^a^ Adjusted for age, sex, history of alcohol, history of smoking, family history of colorectal cancer, exercise, body mass index, hypertension, diabetes melliatus, dyslipidemia, coronary artery disease, peripheral artery disease, cerebrovascular disease, arrhythmia, systolic blood pressure, and diastolic blood pressure.

* *p* < 0.05.

In the univariate analysis, the patients with resting heart rate in the highest quartile (>70 bpm) showed significantly increased odds ratio (OR) of advanced adenoma development, compared to those with resting heart rate in the lowest quartile (≤58 bpm.) (OR: 1.488, 95% CI: 1.202–1.842, *p* < 0.001). After adjusting for age, the patients with resting heart rate in the highest quartile showed a significantly increased OR of advanced adenoma development (OR: 1.489, 95% CI: 1.202–1.844, *p* < 0.001). After adjusting for multiple variables, the patients with resting heart rate in the highest quartile still showed a significantly increased OR of advanced adenoma development (OR: 1.379, 95% CI: 1.099–1.731, *p* = 0.006). Smoking history (OR: 1.287, 95% CI: 1.090–1.520, *p* = 0.003), high BMI of >25 kg/m^2^ (OR: 1.404, 95% CI: 1.190–1.658, *p* < 0.001), and history of DM (OR: 1.391, 95% CI: 1.082–1.788, *p* = 0.010), peripheral artery disease (OR: 4.873, 95% CI: 1.075–22.081, *p* = 0.040), and cerebrovascular disease (OR: 2.896, 95% CI: 1.403–5.979, *p* = 0.004) were also positively associated with an increased risk of advanced adenoma development (**Table 4**). Active exercise was negatively associated with the development of advanced adenoma (OR: 0.804, 95% CI: 0.676–0.957, *p* = 0.014).

**Table 4:** Logistic regression analysis for advanced adenoma development.

Variables	Univariate analysis			Multivariate analysis		
	OR	95% CI	*p value	OR	95% CI	*p value
Age	1.000	0.992–1.008	0.970			
Sex	1.004	0.842–1.197	0.964			
Smoking history	1.305	1.118–1.524	0.001	1.287	1.090–1.520	0.003
Alcohol history	1.043	0.896–1.214	0.587			
Family history of colorectal cancer	1.000	0.724–1.382	1.000			
Active exercise	0.837	0.711–0.986	0.034	0.804	0.676–0.957	0.014
Body Mass Index (≥25kg/m^2^)	1.484	1.262–1.745	<0.001	1.404	1.190–1.658	< 0.001
Hypertension	1.213	1.026–1.434	0.024	1.116	0.932–1.338	0.233
Diabetes Mellitus	1.538	1.218–1.943	<0.001	1.391	1.082–1.788	0.010
Hyperlipidemia	1.144	0.968–1.352	0.115			
Coronary artery disease	1.011	0.775–1.319	0.935			
Peripheral artery disease	6.063	1.354–27.143	0.018	4.873	1.075–22.081	0.040
Cerebrovascular disease	2.585	1.355–4.934	0.004	2.896	1.403–5.979	0.004
Arrhythmia	0.891	0.343–2.316	0.813			
Resting heart rates						
Quartile 1	1.000	Reference		1.000	Reference	
Quartile 2	0.995	0.801–1.236	0.963	0.971	0.773–1.221	0.804
Quartile 3	1.027	0.826–1.277	0.811	0.981	0.779–1.236	0.873
Quartile 4	1.488	1.202–1.842	<0.001	1.379	1.099–1.731	0.006
Systolic blood pressure (mmHg)	1.005	0.998–1.011	0.190			
Diastolic blood pressure (mmHg)	1.002	0.994–1.011	0.599			

*p value when comparing the advanced adenoma group and the non-advanced adenoma group.

OR, odds ratio; CI, confidence interval.

## Discussion

Resting heart rate is known as an independent risk factor for all-cause mortality, cardiovascular mortality, cancer mortality, and CRC-related mortality [6–10]. Resting heart rate is also related to CRC development and advanced adenoma recurrence in CRC patients, as reported in previous studies [11,12,15]. The cross-sectional nature of our study precludes a causal inference between resting heart rate and the risk of developing colorectal adenoma. However, our results might suggest that the high resting heart rate could be implicated in the development of colorectal advanced adenoma through the process of CRC carcinogenesis.

One possible mechanism explaining the decreased risk for advanced adenoma development by active exercise might be the mediating effect of the resting heart rate. Active exercise could increase in resting parasympathetic tone, decrease in response to beta-adrenergic stimulation, and contribute to a decrease in resting heart rates [17]. The most effective way for lowering the resting heart rate is by engaging in regular active exercises, and active exercises could prevent CRC development and recurrence [18,19]. The resting heart rate was significantly lower in the active exercise group than in the sedentary group in the present study. Moreover, there were more active patients in the lower resting heart rate quartiles. Nevertheless, after adjusting for multiple variables, including active exercise, the patients with resting heart rate in the highest quartile still showed significantly increased OR of advanced adenoma development, suggesting that the high resting heart rate is not only a marker of lack of physical activity but also an independent risk factor of advanced adenoma development.

The patients with high resting heart rates were more likely to be smokers. Linneberg et al. demonstrated that smoking heaviness has a causal relationship with a high resting heart rate [20]. Smoking could be the mediating effect of resting heart rate in advanced adenoma development. The mechanism of increased resting heart rate related to smoking is unclear. Nicotine could increase the resting heart rate owing to the release of norepinephrine and epinephrine [21]. Smoking could reflect persistent sympathetic nervous stimulation and lead to autonomic dysfunction [22]. Given that smoking cessation is known to decrease the resting heart rate in a previous study, not only active exercise but also smoking cessation could prevent the development of advanced adenoma by decreasing the resting heart rate [23].

The resting heart rate is significantly associated with the presence of metabolic syndrome [24]. DM, insulin resistance, hyperinsulinemia, and metabolic syndrome have been reported as risk factors of colorectal advanced adenoma and CRC development [25,26]. In our study, the patients with a high resting heart rate showed an increased incidence of DM, increased serum glucose, TG, HbA1C, and insulin levels, and decreased HDL-C levels. The presence of poor metabolic parameters could be related to the increased resting heart rate and advanced adenoma development.

The resting heart rate could be corrected through lifestyle modification, including engaging in active exercises and cessation of smoking, which could eventually improve the metabolic parameters. Many previous studies investigated the effect of beta blockers, because of the weakened norepinephrine signaling in CRC patients. Some studies reported the protective effects of beta blockers in CRC patients [27–29] Beta blockers decreased the proliferation of colorectal cancer cells by inhibiting adrenergic activations [30]. Engineer et al. observed an association between exposure to a combination of angiotensin-converting enzyme inhibitors and beta blockers and increased survival, decreased length of hospitalization, and decreased tumor progression in advanced CRC patients [31]. However, Jansen et al. reported no association between beta blocker use and CRC development after adjusting for covariates in a large population-based CRC cohort [32]. The clinical application of beta blockers as therapeutic agents for CRC is putative, but adrenergic hyperactivity is considered an important risk factor in CRC pathogenesis. Coetho et al. reported that some beta blockers, including propranolol, carvedilol, and atenolol, significantly decreased the proliferation of human CRC cells [30]. In summary, the resting heart rate is a factor that could be modified by improving the lifestyle, such as engaging in aerobic exercises, regular sleep cycle, and smoking and alcohol drinking cessation [33–35]. Furthermore, healthy lifestyle aimed at controlling the resting heart rate could reduce both incidence of and mortality from various metabolic diseases, cardiovascular diseases, advanced adenoma, and CRC.

This study has several limitations. First, we are unable to explain the causal relationship between resting heart rate and advanced adenoma development because of the study’s retrospective, cross-sectional, and case-control design. Second, we only analyzed a single measurement of the resting heart rate and metabolic parameters; we did not perform repeated measurements of these parameters. Nevertheless, we obtained the accurate resting heart rate by 12-lead ECG in a supine position after >10 minutes of sufficient rest in all patients. Third, the measurement of the resting heart rate could be influenced by multiple factors. Fourth, the heart rate variability, linked to cancer prognosis and survival, was not investigated in this study [36,37]. Fifth, we have matched controls based on age and sex, not on potential confounders, such as BMI and smoking. Despite these limitations, the strength of the study is the inclusion of a large number of patients with histologically confirmed colorectal advanced adenomas who underwent health screening examinations; thus, our cohort may be representative of the general population. Moreover, many confounders, such as alcohol and smoking history, obesity, physical activity, and metabolic laboratory data were available.

In conclusion, the resting heart rate was positively associated with the development of advanced adenoma in patients who underwent screening colonoscopy during routine health examinations. Patients with high resting heart rates were more likely to be smokers, to be engaging in less active exercises, and to have DM. We consider that healthy lifestyle modifications aimed at controlling the resting heart rate could decrease the risk of developing advanced adenoma.

## Abbreviations

BMI: body mass index
CI: confidence interval
CRC: colorectal cancer
DM: diabetes mellitus
ECG: electrocardiogram
HbA1C: hemoglobin A1C
HDL: high-density lipoprotein
HR: hazard ratio
HTN: hypertension
IBD: inflammatory bowel disease
LDL: low-density lipoprotein
MMP 6: metalloproteinase 6
OR: odds ratio
PS: propensity score
TG: triglyceride
VEGF: vascular endothelial growth factor
WHO: World Health Organization
